# A novel *CACNA1A* variant in a child with early stroke and intractable epilepsy

**DOI:** 10.1002/mgg3.1383

**Published:** 2020-07-21

**Authors:** Franciska J. Gudenkauf, Mahshid S. Azamian, Jill V. Hunter, Anuranjita Nayak, Seema R. Lalani

**Affiliations:** ^1^ School of Medicine Baylor College of Medicine Houston TX USA; ^2^ Department of Molecular and Human Genetics Baylor College of Medicine Houston TX USA; ^3^ Department of Radiology Baylor College of Medicine Houston TX USA; ^4^ Section of Pediatric Neurology and Developmental Neuroscience Department of Pediatrics Baylor College of Medicine Houston TX USA

**Keywords:** CACNA1A, epilepsy, stroke

## Abstract

**Background:**

*CACNA1A* variants have been described in several disorders that encompass a wide range of neurologic phenotypes, including hemiplegic migraine, ataxia, cognitive delay, and epilepsy. To date, ischemic stroke caused by a *CACNA1A* variant has only been reported once in the literature.

**Methods:**

We describe a 4‐year‐old female with recurrent ischemic strokes beginning at 6 weeks of age, intractable epilepsy, and significant global developmental delay. Exome sequencing (ES) was completed for her evaluation.

**Results:**

We found a novel de novo, likely pathogenic variant, p.Leu1692Gln in *CACNA1A* by ES. The substitution affects a leucine residue that is highly conserved in species from fish to primates.

**Conclusion:**

We present the second case of recurrent ischemic strokes in a patient with *CACNA1A* mutation. Our findings expand the phenotypic heterogeneity related to Ca_v_2.1 (P/Q*‐*type) calcium channel dysfunction and suggest consideration of *CACNA1A* disorder in evaluation of pediatric strokes.

## Graphical abstract

We describe a four‐year‐old female with recurrent ischemic strokes beginning at six weeks of age, intractable epilepsy, and significant global developmental delay, who was found to have a novel de novo likely pathogenic p.Leu1692Gln variant in CACNA1A. Our report presents the second described case of recurrent ischemic strokes in a patient with CACNA1A mutation and expands the phenotypic heterogeneity related to variants in this gene.

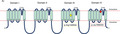

## 1 INTRODUCTION

**Figure 1:**
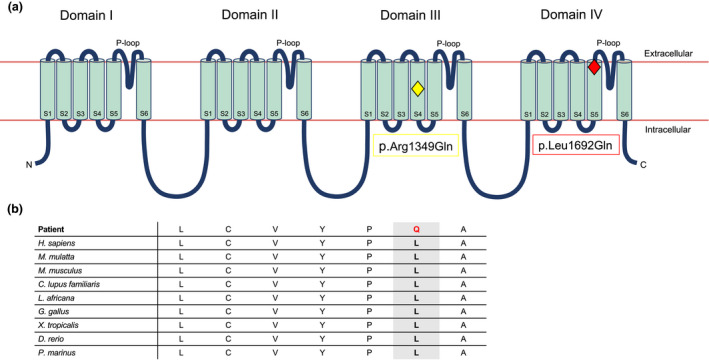
Structure of the alpha‐1 pore‐forming subunit of the Ca_v_2.1 (P/Q‐type) voltage‐gated calcium channel. (a) Secondary structure; our patient's variant, p.Leu1692Gln, is located in the S5 segment of domain IV (red diamond), while the single previously reported stroke‐associated variant, p.Arg1349Gln, is located in the S4 segment of domain III (yellow diamond). (b) Amino acid conservation across species; the leucine residue (L) at 1692 codon is highly conserved across species. Our patient's variant (p.Leu1692Gln) substitutes a glutamine residue (Q) instead

The *CACNA1A* gene (OMIM #601011) codes for the alpha‐1 pore‐forming subunit of the P/Q‐type voltage‐gated calcium channel, which is located in the neuronal membrane (Currie, 2010; Luo et al., 2017). The Ca_v_2.1 (P/Q‐type) channels are expressed in mammalian brain and are responsible for Ca^2+^ influx to modulate membrane excitability, synaptic transmission, and calcium‐dependent gene transcription (Takahashi & Momiyama, 1993; Westenbroek et al., 1995). The alpha‐1 subunit encoded by *CACNA1A* contains four repeated domains (I–IV), each consisting of six membrane‐spanning segments (S1–S6) and a P‐loop between S5 and S6 (Currie, 2010); the four S4 segments constitute the voltage sensor of the channel and the S5 and S6 segments along with the P‐loops form the pore lining (Figure 1a). Pathogenic variants in *CACNA1A* can lead to a variety of neurological symptoms and several well‐described disorders (Byers, Beatty, Hahn, & Gospe, 2016; Luo et al., 2017). Familial hemiplegic migraine type 1 (FHM1, OMIM #141500), caused by gain‐of‐function variants in *CACNA1A*, is an extreme type of migraine with aura that typically presents in the first or second decade of life with episodes of headaches, sensory loss, visual disturbance, hemiparesis, and cerebellar signs such as nystagmus or ataxia (Byers et al., 2016; Jen, 2001). Episodic ataxia type 2 (EA2, OMIM #108500) is usually due to loss‐of‐function variants that lead to paroxysms of ataxia, vertigo, and nausea typically lasting minutes to days (Spacey, 2003). Individuals with EA2 may also experience migraines and hemiplegia like those with FHM1 (Spacey, 2003). Abnormal trinucleotide repeat expansion in the *CACNA1A* gene causes spinocerebellar ataxia type 6 (SCA6, OMIM #183086), which normally presents in adulthood with progressive gait ataxia, incoordination, tremors, dysarthria, and nystagmus (Case y & Gomez, 1998).

Although these disorders are caused by different pathogenic variants, it is evident that phenotypic features are shared among them (Byers et al., 2016). Even family members with the same variant may exhibit variable expressivity anywhere within, or even outside, the spectrum of symptoms associated with these disorders (Byers et al., 2016). In addition to these three disorders, *CACNA1A* variants have been associated with epilepsy and several other neurologic manifestations, including global developmental delay, hypotonia, and cerebellar hypoplasia, highlighting the incredibly diverse variability in phenotype resulting from genetic alterations in this gene (Byers et al., 2016; Liu et al., 2018; Luo et al., 2017). Indeed, with the commonplace use of molecular genetic testing, new associations between *CACNA1A* variants and phenotypic manifestations will surely continue to be revealed.

Here, we present the case of a 4‐year‐old female with a history of recurrent ischemic strokes starting at 6 weeks of age, intractable epilepsy, and significant global developmental delay who was found to have a novel likely pathogenic, de novo variant in *CACNA1A* (p.Leu1692Gln). To our knowledge, there has only been one previously reported case of ischemic stroke caused by *CACNA1A* mutation (p.Arg1349Gln) in a young girl with FHM1 (Knierim et al., 2011). However, the particular variant in the *CACNA1A* gene identified in our patient has not yet been described.

## 2 METHODS

### 2.1 Editorial policies and ethical considerations

The study was approved by the ethics committee at Baylor College of Medicine. Informed consent was obtained from the family for inclusion in the study.

### 2.2 Case report

**Figure 2:**
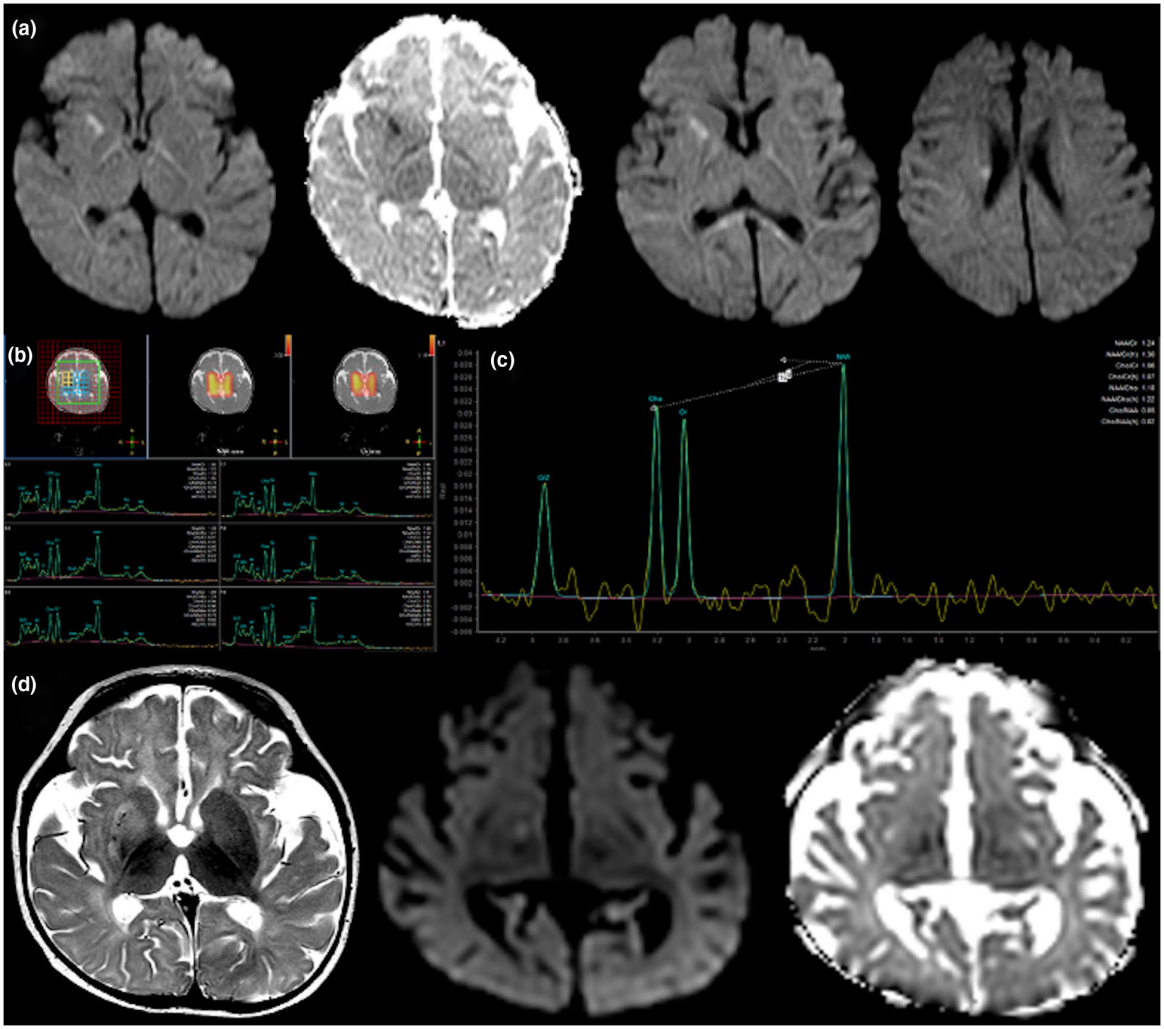
Select brain imaging in a patient with p.Leu1692Gln *CACNA1A* variant, recurrent strokes, and intractable epilepsy. (a) MRI at 8 weeks of age; iso DWI and ADC maps demonstrating restricted diffusion‐weighted imaging returned from the right anterior putamen, posterior limbs of the internal capsule bilaterally, right corticospinal tract, and splenium of the corpus callosum, consistent with acute right putaminal infarction and postictal changes. (b) H1 MRS short‐TE multivoxel at 3.5 months of age and (c) H1 MRS long‐TE single‐voxel at 3.5 months of age; placed in right basal ganglia, both demonstrating a relative paucity of NAA without evidence for lactate doublet. Appearance is consistent with neuronal and axonal loss. (d) MRI at 5.5 months of age; axial T2‐weighted and axial iso DWI and ADC maps through the plane of the basal ganglia show increased T2 hyperintensity returned from the right basal ganglia with new restricted diffusion abnormality in the right‐sided globus pallidus. Appearances consistent with new right globus pallidus infarction within the preceding 7–10 days. There is bilateral but symmetric underopercularization noted, together with mild right posterior plagiocephaly. ADC, apparent diffusion coefficient; DWI, diffusion‐weighted restriction; MRI, magnetic resonance imaging; MRS, magnetic resonance spectroscopy; NAA, N‐acetylaspartate; TE, echo time

We describe a 4‐year‐5‐month‐old Hispanic female who was born to non‐consanguineous parents. Her early medical history was seemingly unremarkable until about 6 weeks of age, when she presented with transient tonic stiffening of her left upper extremity without any alteration in consciousness, in the setting of fever. She then presented 2 weeks later with an atypical febrile seizure involving her left hemibody, followed by developmental regression with loss of visual tracking. Her head circumference was noted to be normal at 2 months of age (39 cm). Electroencephalogram (EEG) revealed corroborating epileptiform activity from the right centro‐parietal regions. Magnetic resonance imaging (MRI) showed multifocal restricted diffusion abnormality in the right anterior putamen, bilateral posterior limbs of the internal capsule, right corticospinal tract, and splenium of the corpus callosum, consistent with recent right putaminal infarction (Figure 2a). Magnetic resonance arteriography (MRA) and magnetic resonance venography (MRV) were normal. Hypercoagulability workup was negative except for a transiently positive lupus anticoagulant. Echocardiogram showed a small‐ to moderate‐sized fenestrated secundum atrial septal defect versus stretched patent foramen ovale (PFO). She was treated with daily aspirin and discharged home on levetiracetam.

The patient presented again at 3.5 months of age with fever and status epilepticus. EEG showed diffuse background slowing with periods of generalized suppression, focal voltage attenuation in the left hemispheric leads, and multifocal epileptiform discharges. On brain imaging, there was interval evolution of infarction in the right putamen with resolution of restricted diffusion. Magnetic resonance spectroscopy (MRS) at the time showed a relative paucity of N‐acetylaspartate without lactate doublet, consistent with neuronal and axonal loss (Figure 2b,c). Around 4 months of age, she was diagnosed with an apparently spontaneous right internal jugular vein nonocclusive thrombus and was treated with enoxaparin. Despite adequate anticoagulation, she presented at 5.5 months of age with increasing seizures and new diffusion restriction on brain MRI involving the right globus pallidus concerning for a new infarct (Figure 2d). She had repeated hospitalizations due to frequent refractory status epilepticus, developed intractable epileptic encephalopathy, and was implanted with a vagus nerve stimulator by age 8 months due to medical refractoriness. Her seizure management, complicated by apneic events, required repeated intubations, eventually leading to tracheostomy. Brain MRI at 7 months of age showed no new areas of diffusion restriction with interval resolution of the previously noted changes. However, there were prominent ventricles and sulci suggestive of diffuse cerebral volume loss. At 4 years and 5 months of age follow‐up visit, she was nonambulatory, nonverbal, and minimally interactive. She was gastrostomy tube‐dependent. Her physical exam revealed acquired microcephaly with head circumference of 46.7 cm (*Z* = −2.05), spasticity of the lower extremities, increased deep tendon reflexes, and axial hypotonia. Her seizures had improved with her current treatment regimen and no new neurologic deficits were reported.

### 2.3 Diagnostic Workup

Extensive diagnostic workup was pursued throughout this time, with the majority of studies resulting as either negative or inconclusive. Newborn screenings, comprehensive chromosomal microarray, and mitochondrial DNA next generation sequencing results were all normal. Tests for inborn errors of metabolism were nondiagnostic (e.g., acylcarnitine profile, plasma amino acids, urine organic acids, and blood lactate levels). Global Metabolomics Assisted Pathway Screen and cerebrospinal fluid neurotransmitter studies were normal. Cerebrospinal fluid infectious workup was negative, as were laboratory tests for arginine:glycine amidinotransferase deficiency. Muscle biopsy was done at age 7 months, which showed a paucity of mitochondria suggesting mitochondrial depletion. Electron transport chain study indicated a reduction in several respiratory chain complex activities; however, the findings did not satisfy a minor criterion of the modified Walker criteria for the diagnosis of a respiratory chain disorder. To obtain a definitive diagnosis, trio exome sequencing was performed.

## 3 RESULTS

Trio exome sequencing identified a heterozygous, likely pathogenic de novo variant, c.5075T>A (p.Leu1692Gln) in *CACNA1A* (NM_001127221.1; chr19:13346084). This substitution (leucine to glutamine) affects a leucine residue that is highly conserved in species from fish to primates (Kent et al., 2002) and is predicted to be damaging by in silico prediction programs (Figure 1b). This variant is not observed in gnomAD (Karczewski et al., 2020). A heterozygous variant of unknown clinical significance, c.5900G>A (p.Arg1967Gln) was also seen in *CACNA1A* (NM_001127221.1; chr19:13325090), inherited from her asymptomatic father. This variant was previously reported in a cohort study for risk assessment in epilepsy (Klassen et al., 2011), but has also been observed in gnomAD. It has been classified as benign or likely benign in ClinVar by multiple submitters (Landrum et al., 2018).

## 4 DISCUSSION

Here, we present a young child with early ischemic stroke, intractable epilepsy, and significant global developmental delay due to a novel de novo variant, p.Leu1692Gln in *CACNA1A*. As discussed, variants in this gene have been implicated in a wide range of neurologic phenotypes and well‐described disorders, such as FHM1, EA2, and SCA6 (Byers et al., 2016). In recent years, *CACNA1A* has become associated with a multitude of neurological phenotypes, including congenital hypotonia, cerebellar hypoplasia, cognitive delay, refractory epilepsy, and stroke (Klassen et al., 2011; Knierim et al., 2011; Liu et al., 2018). To our knowledge, this is only the second case of stroke described in a patient with a *CACNA1A* variant. In 2011, Knierim et al. reported a 6‐year‐old female with a diagnosis of FHM1 who presented with recurrent ischemic strokes after minor head trauma, associated with seizures and other neurologic symptoms, who was found to have a novel p.Arg1349Gln variant in *CACNA1A*. This variant is located in the helical region of the S4 segment in domain III and was hypothesized to be a gain‐of‐function mutation, whereas our patient's variant is in the helical region of the S5 segment in domain IV (Knierim et al., 2011; The UniProt Consortium, 2019; Figure 1a). FHM1 is a severe form of migraine with aura, which has been independently associated with a risk for both stroke and epilepsy (Jen, 2001; Kurth, Chabriat, & Bousser, 2012; Rogawaski, 2012). Migraine with aura doubles the risk of ischemic stroke, likely due to cortical spreading depression (CSD) which triggers headache and reduces cerebral blood flow to a level that induces ischemia (Jolobe, 2012; Kurth et al., 2012). Specifically, in FHM1, ion homeostasis is disrupted due to the calcium channel defects, which increases neuronal excitability and lowers the threshold for CSD (Kurth et al., 2012). In transgenic mouse models of FHM1, the calcium channels open with smaller depolarizations and stay open for a long time, thus, increasing neuronal excitability with glutamate release after continued presynaptic influx of calcium (Tottene et al., 2005). It has also been shown that *Cacna1a* transgenic mice required increased cerebral blood flow and suffered stroke even with milder ischemia, developed larger infarcts, and experienced more severe outcomes (Eikermann‐Haerter et al., 2012; Jolobe, 2012). Thus, the increased susceptibility to ischemia secondary to reduced cerebral blood flow from CSD may explain the association between migraine and stroke (Kurth et al., 2012). Additionally, the neuronal excitability that precedes CSD is implicated in seizure initiation (Nye & Thadani, 2015; Rogawaski, 2012); further, epilepsy can frequently present with peri‐ictal migrainous headaches, and migraine may trigger epilepsy, or even occur simultaneously. CSD is implicated as an important mechanism in all three conditions––a mechanism disrupted by *CACNA1A* mutation (Nye & Thadani, 2015).

Our patient presented very young at 6 weeks of age with stroke. Thus, it remains unknown if her phenotype would be more similar to classic FHM1 if she had not had a stroke at such a young age. The question also remains if this novel variant predisposes to early infarcts after minor trauma/fever, as our patient was febrile on both occasions when she presented at 6 and 8 weeks of age. Overall, her phenotype appears to be on the severe end of *CACNA1A*‐related disease. Yet, it is uncertain whether her clinical course stems from the sequelae of stroke, the sequelae of refractory epilepsy, the underlying novel *CACNA1A* variant, or, most likely, a combination of all of these. It also bears questioning whether the stroke can be fully attributed to the underlying genetic variant, or to some comorbid process that has now complicated her clinical course. It is interesting that similar to other metabolic strokes, such as seen in cerebral autosomal dominant arteriopathy with subcortical infarcts and leukoencephalopathy (CADASIL) and mitochondrial encephalopathy, lactic acidosis, and stroke‐like episodes (MELAS), the distribution of the infarcts in her does not correspond to vascular territories. Other thromboembolic possibilities were excluded by her normal MRA and MRV studies. For the same reason, the ASD versus PFO found on echocardiogram was unlikely to be implicated in her stroke. Along with MRS lacking a lactate peak, other workup was nondiagnostic for a mitochondrial disease. Coagulopathy was ruled out, which can also predispose to arterial ischemic strokes (Felling, Sun, Maxwell, Goldenberg, & Bernard, 2017). Thus, based on her extensive nondiagnostic workup and a previously reported individual with FHM1 and stroke related to *CACNA1A* variant (Knierim et al., 2011), we propose that the de novo variant in this gene in our patient is most likely the cause of her recurrent strokes.

While early recurrent strokes caused significant morbidity in our patient, the refractory epilepsy that developed later resulted in more severe neurological sequelae. Our patient's initial MRI showed diffusion restriction in the right anterior putamen and right corticospinal tract, with EEG revealing epileptiform activity from the right centro‐parietal regions; thus, it is conceivable that the seizure activity was related to ischemia. However, repeat EEG at 3.5 months after intractable seizures showed multifocal epileptiform discharges and voltage attenuation in the left hemisphere, despite all prior ischemic insults having affected the right side; this may suggest the seizures are not fully attributable to a history of stroke and instead may indicate progression to epileptic encephalopathy. Further, seizures, epilepsy, and epileptic encephalopathy all have been described as part of the phenotypic spectrum of *CACNA1A* mutations (Byers et al., 2016). In the previously described case of stroke due to *CACNA1A* mutation, the affected patient also presented with seizures (Knierim et al., 2011). Thus, it is possible that our patient's epilepsy represents phenotypic manifestations of her underlying variant, as opposed to being fully caused by ischemic insults. Further, our patient also manifested a p.Arg1967Gln variant of unknown clinical significance, which has been reported in a study evaluating risk assessment in epilepsy (Klassen et al., 2011), but also previously classified as benign (Landrum et al., 2018). It is unclear if this variant is also contributing to her phenotype. Additionally, our patient's final MRI at 7‐months showed diffuse cerebral volume loss, likely not fully attributable to stroke; this manifestation, as well as her acquired microcephaly, may also be phenotypic of the variant, as decreased brain volume such as cerebellar hypoplasia has previously been reported (Luo et al., 2017).

In summary, we report a second patient with recurrent strokes related to *CACNA1A* mutation. Our findings expand the phenotypic heterogeneity related to Ca_v_2.1 (P/Q‐type) calcium channel dysfunction and suggest consideration of *CACNA1A* disorder in evaluation of pediatric strokes, especially if the brain lesions do not conform to vascular territories. While we postulate a gain‐of‐function mechanism for this Ca_v_2.1 voltage‐gated Ca^2+^ channel in causing stroke in our patient, further studies are needed to understand the basis of stroke related to the p.Leu1692Gln variant and determine the specific impact of this variant on *CACNA1A* function.

## CONFLICT OF INTEREST

The authors declare no conflict of interest related to the manuscript.

## AUTHOR CONTRIBUTIONS

F.J.G. and S.R.L. planned the report. M.S.A. consented and enrolled the patient/family in the study. S.R.L., A.N., and J.V.H. provided the clinical data. F.J.G. and J.V.H. created the figures. F.J.G., S.R.L., and A.N. drafted the manuscript. All coauthors read and approved the final manuscript as submitted.

## Data Availability

Variant was submitted to the National Center for Biotechnology Information ClinVar database (https://www.ncbi.nlm.nih.gov/clinvar/).
